# Structuring communication relationships for interprofessional teamwork (SCRIPT): a cluster randomized controlled trial

**DOI:** 10.1186/1745-6215-8-23

**Published:** 2007-09-18

**Authors:** Merrick Zwarenstein, Scott Reeves, Ann Russell, Chris Kenaszchuk, Lesley Gotlib Conn, Karen-Lee Miller, Lorelei Lingard, Kevin E Thorpe

**Affiliations:** 1 Keenan Research Centre in the Li Ka Shing Knowledge Institute, St. Michael's Hospital, 30 Bond Street, Toronto, Canada, M5B 1W8; 2 Department of Health Policy, Management and Evaluation, Faculty of Medicine, University of Toronto, 155 College Street, Suite 425, Toronto, Canada, M5T 3M6; 3 Sunnybrook Health Sciences Centre Research Institute, 2075 Bayview Avenue, Toronto, Canada, M4N 3M5; 4 Centre for Faculty Development, St. Michael's Hospital, 30 Bond Street, Toronto, Canada, M5B 1W8; 5 Wilson Centre for Research in Education, University of Toronto and University Health Network, 200 Elizabeth Street, 1ES-565, Toronto, Canada, M5G 2C4; 6 Office of Interprofessional Education, University of Toronto and University Health Network, 750 Dundas Street West, Suite 3-302, Toronto, Canada, M6J 3S3; 7 Department of Paediatrics, Faculty of Medicine, University of Toronto; 8 Department of Public Health Sciences, Faculty of Medicine, University of Toronto, 155 College Street, 6^th ^Floor, Toronto, Canada, M5T 3M7

## Abstract

**Background:**

Despite a burgeoning interest in using interprofessional approaches to promote effective collaboration in health care, systematic reviews find scant evidence of benefit. This protocol describes the first cluster randomized controlled trial (RCT) to design and evaluate an intervention intended to improve interprofessional collaborative communication and patient-centred care.

**Objectives:**

The objective is to evaluate the effects of a four-component, hospital-based staff communication protocol designed to promote collaborative communication between healthcare professionals and enhance patient-centred care.

**Methods:**

The study is a multi-centre mixed-methods cluster randomized controlled trial involving twenty clinical teaching teams (CTTs) in general internal medicine (GIM) divisions of five Toronto tertiary-care hospitals. CTTs will be randomly assigned either to receive an intervention designed to improve interprofessional collaborative communication, or to continue usual communication practices.

Non-participant naturalistic observation, shadowing, and semi-structured, qualitative interviews were conducted to explore existing patterns of interprofessional collaboration in the CTTs, and to support intervention development. Interviews and shadowing will continue during intervention delivery in order to document interactions between the intervention settings and adopters, and changes in interprofessional communication.

The primary outcome is the rate of unplanned hospital readmission. Secondary outcomes are length of stay (LOS); adherence to evidence-based prescription drug therapy; patients' satisfaction with care; self-report surveys of CTT staff perceptions of interprofessional collaboration; and frequency of calls to paging devices. Outcomes will be compared on an intention-to-treat basis using adjustment methods appropriate for data from a cluster randomized design.

**Discussion:**

Pre-intervention qualitative analysis revealed that a substantial amount of interprofessional interaction lacks key core elements of collaborative communication such as self-introduction, description of professional role, and solicitation of other professional perspectives. Incorporating these findings, a four-component intervention was designed with a goal of creating a culture of communication in which the fundamentals of collaboration become a routine part of interprofessional interactions during unstructured work periods on GIM wards.

**Trial registration:**

Registered with National Institutes of Health as NCT00466297.

## Background

Interprofessional education has been promoted internationally [1,2] and nationally [3,4] by policy makers as a means to improve collaboration and service delivery. It is often argued that if individuals from different professions learn together their professions will collaborate more effectively, improving care and the delivery of service. This argument has strong appeal, and as a result, there has been a steady growth of interprofessional education within the health and social care systems in the United Kingdom, the United States, continental Europe, Australia and more recently Canada [5]. However, findings from systematic reviews continue to report that there is only limited evidence that interprofessional education has an impact on improving interprofessional collaboration, patient care, patient-centredness or patient outcomes [6-8].

Funded by Health Canada, the SCRIPT Programme (Structuring Communication Relationships for Interprofessional Teamwork [9]) seeks both to advance the evidence base on interventions to achieve interprofessional education for collaborative patient-centred practice (IECPCP) and to achieve sustainable transformation in the conduct, learning and evaluation of interprofessional teamwork in the Toronto Academic Health Sciences Network (TAHSN), the partnership between the University of Toronto and its fully affiliated health care institutions.

### Creating a culture of collaborative communication

We developed an intervention with an aim of creating a culture of *interprofessional collaborative communication*. Whereas communication most generally is the exchange of information between individuals, our goal is to encourage the kind of communication that fosters collaborative working relationships between professionals. The intervention is designed to promote the kind of communicative exchange that would encourage joint problem solving and provision of excellent interprofessional patient-centred care. Despite the importance of information exchange between professionals, our emphasis is on what comes next: putting the information together to arrive at joint solutions and deeper understandings.

We assume that when a professional provides another with a plan or problem and has the opportunity to explicate or query a colleague's point of view, opportunities for new and more effective ideas or solutions emerge. Here, we subscribe to the principle that 'the whole is greater than the sum of its parts.' When collaborative communication is effective, the yield for the patient will be greater than if a single professional manages their care in isolation.

Collaborative communication in our model is both intentional and opportunistic. It is purposeful as health care professionals will determine the context in which the collaborative communication exchange is warranted and with whom. At minimum, one other person (from another profession) is required to make this fit the criterion of 'interprofessional.' However there is no upper limit on the number of professionals, and indeed patients or family members may be included in the collaborative exchange. The main criterion guiding whether or not professionals choose to seek out input from their colleagues is that without collaborative communication, the problem could not be solved uniprofessionally. The purpose and context of the collaborative communication are entirely decided by the professionals engaged in the activity.

The general internal medicine (GIM) project has investigated interprofessional behaviours in hospital clinical teaching teams (CTTs) by using naturalistic observations and in-depth interviews. These data were foundational for the design of a workplace-based staff intervention that is intended to promote interprofessional collaborative communication.

There were two important findings from our preparatory qualitative observations of ward interactions. First, there is a high turnover rate of the medical staff on GIM wards due to their professional rotations. Turnover presents a major challenge for both clinical (medicine, nursing, other health professionals) and administrative staff and trainees in relation to learning the names, roles, and scopes of practice of individuals working beside them [10]. Second, GIM teams have distinctive norms and established mechanisms for facilitating formalized and planned interprofessional communication and collaboration, such as daily interdisciplinary rounds. However, none of the GIM divisions we observed has developed a normative procedure for interprofessional communication and collaboration during *unstructured*, *unscheduled *work periods.

Moreover, our research showed that important characteristics of communication and collaboration behaviours are problematic in *opportunistic *encounters. Four areas were notable:

1. Mutual interpersonal knowledge of given names and surnames is often absent. Staff members perceive that they do not know very many others' names, and that their own names are usually unknown to others;

2. Mutual interpersonal knowledge of another's occupational title, professional role, or educational credentials is absent or ambiguous;

3. Interprofessional patient-related interactions are not commonly marked by sharing of unique, profession-specific knowledge bases, e.g., care-plan activities or diagnostic questions; clinical health management concerns are sequestered;

4. Interprofessional patient-related interactions pass information along routes that are seemingly one-way, unidirectional pathways; there is little reciprocity.

Hence, the intervention design focuses on improving interprofessional communication and collaboration outside of structured meetings, specifically to increase the quality of opportunistic encounters between professionals.

A cluster randomized design is the only possible trial design in our setting. Department of Medicine managers and nursing and professional leaders organize staff members into functional GIM teams. We can not define teams as intervention or control teams and then randomize individual staff members to teams; randomization at the individual level would require us to deconstruct and re-construct teams according to random assignments, an impossible process. Individual randomization to intervention status without team randomization would result in contamination as intervention group staff members worked alongside control group staff members.

## Methods

### Participants and setting

General internal medicine is organized as a 'division' in hospitals of the TAHSN, housed within Departments of Medicine. In many hospitals, GIM divisions have long been organized into groups known as clinical teaching teams. Teams serve goals related to patient care, education, collegiality, and administration.

GIM divisions in TASHN hospitals are composed of between 200 and 300 staff and students. The medical staff of a CTT is composed of a staff physician, medical residents, and clinical clerks. The number of medical trainees working in a clinical teaching team varies at any particular time between about five and nine. Usually, it includes one senior resident, a second- or third-year post-licensure medical resident (PGY2), two first-year medical residents (PGY1), one pre-licensure medical student in the fourth year of medical school (fourth-year clinical clerk), and two or three pre-licensure medical students in the third year of medical school (third-year clinical clerks).

Medical teams work with groups of non-physician health professionals. In Ontario, Canada, these are full-time, part-time, temporary, or casual employees of the hospital. The GIM CTTs in our trial employ professionals in all of these statuses and they may or may not work exclusively in wards of the GIM division. A typical team may include a variety of professions, most commonly nursing, physical therapy, occupational therapy, social work, diet and nutrition, and pharmacy. In the TAHSN system, the non-medical, non-nursing professions have traditionally been called 'allied health professions' and their individual practitioners are known as 'allied health professionals,' or 'allied staff.' These professions and their members are regulated under the Regulated Health Professions Act of the province of Ontario and their respective disciplinary colleges are licensed by laws of the province of Ontario, Canada.

The SCRIPT study is expected to operate in twenty medical teams making up the GIM divisions of five TAHSN hospital sites. Inclusion criteria for hospitals are: the hospital corporation or its site(s) must be a member of the TAHSN and the site must have a general internal medicine service within its Department of Medicine. A medical team is eligible if it has a regularly scheduled rotation of attending staff physicians and residents; has both nursing and allied health professional staff members working with it regularly; and is not a specialized team providing devoted care to particular medical or surgical cases or conditions, e.g., stroke.

### Intervention

The intervention is a four-step collaborative communication protocol, a quasi-script for face-to-face, collaborative interprofessional interaction. We will ask GIM staff members and trainees to use the four following steps when they have face-to-face patient-related interactions with individuals whom they know (or believe) are members of a different professional group than their own:

1. Name – Introduce oneself to the member(s) of the other profession by name;

2. Role – State to the other interactant(s) one's own professional role in the team or GIM division and describe it with respect to the target patient under discussion;

3. Issue – Share with the member(s) of the other profession one's unique, profession- and training-specific issues, problems, or plans relating to the target patient under discussion; and

4. Feedback – Elicit interaction-specific feedback from the other participant(s) in the interaction by using prompts such as, "Do you have any concerns?" or, "Is there something else I should consider?"

These four steps illustrate our approach to two key intervention goals. First, by continuously introducing oneself by name and role in relation to a specific patient, the typical social barriers in a rapidly changing GIM division – role confusion and anonymity – are reduced. Second, by sharing one's professional perspective and eliciting a collegial point of view, opportunities to solve a patient care problem and/or extend care beyond the limits of uniprofessional practice are elevated.

For GIM medical staff and trainees, intervention implementation will begin in the orientation of a new rotation of medical residents by a team's attending staff physician. This orientation is regularly scheduled during the first week of the rotation. We will enlist the attending physician of each GIM division's two intervention-group medical teams to explain and promote the importance of each of the above mentioned communication behaviours to his or her resident team physicians. Likewise, the same promotional activity will be offered by other professional staff leaders in the intervention CTTs to the professionals who report to them, nurses and allied health professionals. For all professional leaders there will be frequent, opportunistic reinforcements in formal or informal meetings, in one-to-one or group uniprofessional or interprofessional settings.

Implementation will be augmented in the following ways. First, we will establish a broad network of core "faculty advocates" in the intervention teams. Advocates will act as role models and provide direct verbal instruction to their colleagues on the team. We will ask the Patient Care Managers (or equivalents) on intervention teams to enlist the support of staff members who are willing to champion the intervention. Second, advocates will receive initial training about the intervention etiquette from research staff. Third, researchers will provide consultation to advocates to support them in the training, implementation, and uptake goals. Finally, we will use email as a delivery channel to inform advocates of the latest research results and to offer suggestions for reinforcement throughout the duration of the study. The responsibility for implementation of the intervention therefore is shared by researchers and staff-participants in GIM. We anticipate that these combined initiatives will promote increased buy-in and uptake among front line staff-participants in the GIM experimental teams.

In each intervention CTT we expect that thirty minutes will be required to explain the intervention to professional leaders and to field questions. We expect it will take ten minutes of discussion by each of the professional leaders with their trainee or reporting colleagues in each profession. We expect that the time cost of reinforcement will be fleeting, and might occur two or three times per week, at such times as regular rounds or meetings within professions, in interprofessional team meetings, in individual supervision and education meetings, and in opportunistic conversations.

The intervention will run approximately eight weeks in the intervention CTTs. In this time it will expose two rotations of staff and resident physicians and the other health professional staff with whom they work on the teams to the communication and collaboration script. At each hospital site, staff and patient outcomes related to each of the two intervention and control health care teams will be compared.

### Qualitative data collection

The use of qualitative methods to support the development and evaluation of RCTs for complex interventions to improve health, including observational ethnographic research and individual in-depth interviews, has been promoted by the UK Medical Research Council [11]. Non-participant ethnographic research [12,13] characterized by focused, short-term field observations [14], shadowing [15], and qualitative, semi-structured interviews will be conducted during the intervention implementation period by experienced qualitative researchers.

Researchers will position themselves unobtrusively throughout the wards during brief, focused field visits and will collect descriptive and reflective data on verbal and non-verbal interprofessional interactions. Descriptive observation notes will report as closely as is possible the verbatim conversations and sequences of activities that occur. They will identify those present only by professional or occupational role (i.e., no names or other identifiers will be used). Reflective notes will record the researcher's attributions of meanings of the observed interactions.

Shadowing will occur with a purposive sample of GIM CTT staff from a range of professions (e.g., social workers, staff physicians, registered nurses) and training levels (e.g., pre-licensure physiotherapy students, post-licensure medical students). The number of shadowed participants will be 6–7 participants per team, an adequate number to achieve saturation within a homogenous subgroup [16]. Shadowing will involve recording for continuous one-hour periods the behaviours, interactions, and activities of the participant in public areas where patient care is organized or discussed; the researcher will not enter patients' rooms and will avoid observing and recording patient-healthcare provider interactions that could occur outside of rooms, e.g., in halls, lounges, and other common areas.

On the ward floors, there will be no observation-related interruptions to team staff and they will experience no time cost. Shadowing will be arranged with staff members based on mutual availability and convenience, usually between 8:00 a.m. and 5:00 p.m.

During the shadowing sessions, the researcher will ask the participant to explain what he or she is doing in order to prompt not only a running commentary of the particular task being performed, but the social and organizational reasons for the behaviour [15]. The researcher will also attend to the presence and effect of the four steps of the intervention within each interprofessional interaction observed during the shadow period.

Interviews will be done with a purposive sample of GIM CTT staff. The intent is to more fully understand the culture of interprofessional communication among the specific teams, to further explore phenomena identified during observations or shadow periods, and to understand and document activities related to the adoption of the intervention and changes in practice. Interviews will be semi-structured and will be initially developed from the existing literature on interprofessional education. However, interviews will be individualized to allow the researcher to posit questions based on the ongoing observational and shadowing data, as well as to follow up on spontaneous participant comments during interview. Interviews will continue until researchers determine that saturation has been reached, that is, until researchers feel that no new information will be garnered through further interviews [16,17].

The use of qualitative observational and interview techniques will enable researchers to document and monitor complex interactions between the intervention, its settings, and its adopters [18]. In addition, direct observation produces rigor when combined with other methods, for example, it can illuminate discrepancies between participants' quantitative and qualitative self-reports of interprofessional collaborative communication practices and their actual communication and interaction patterns [19].

### Ethics oversight and informed consent

The SCRIPT GIM project has three phases: (1) ethnographic observation and intervention design, (2) pilot study, and (3) cluster randomized controlled trial. Ethics approval will be requested at all participating hospitals and the University of Toronto research ethics boards for each phase. To date, approval was given by three hospitals and the university for phase 1 ethnographic observations and intervention design. Approval was granted by one hospital and the university for the phase 2 pilot study. We received ethics approval from two hospitals and the university to conduct phase 3, the cluster RCT.

Notices will be posted describing the presence and operation of a research program in GIM. The study will be described by the senior hospital staff from each profession in each clinical teaching team at scheduled staff meeting times like bullet rounds and Kardex. They will inform staff members of the presence of the SCRIPT researchers in the GIM teams and indicate that staff members who do not wish to be a subject of research observations can make such a declaration to SCRIPT's observers. Subsequently, observers will not approach these individuals during shadowing sessions and will not record any observations about them. Staff members will not be shadowed into patient rooms and no identifiable patient information will be recorded. Fieldwork observers will not attempt to gain access to any written, identifiable personal health information.

### Objectives: hypotheses

The SCRIPT Programme aims to create a cultural shift in the way health professionals communicate and collaborate in GIM hospital teams. There are three objectives for the SCRIPT Programme, the first two of which have been successfully completed. They are:

1. To assess interprofessional communication and collaboration;

2. To create an intervention – a tool or other process – that improves interprofessional communication and collaboration;

3. To implement and evaluate the effects of the intervention on outcomes.

The objective of the trial is to implement and evaluate the effects of an intervention aimed at enhancing collaborative communication between professionals and improving patient-centred care.

The research hypotheses for trial-related quantitative outcomes are:

1. Patients who are discharged from intervention teams will be less likely to be readmitted to hospital than patients discharged from control teams;

2. Mean time to readmission will be longer for patients discharged from intervention teams than for patients discharged from control teams;

3. Patients who are cared for by intervention teams will have shorter mean lengths of stay than patients in control teams;

4. Survey-measured perceptions of interprofessional collaboration will be higher among staff members of intervention teams than staff members of control teams;

5. Patients will report higher satisfaction with care after being treated by intervention teams than by control teams;

6. There will be an association between the number of calls to staff members' paging devices and team status; intervention-team staff members will receive fewer pages;

7. Adherence rates to evidence-based best practices for prescription drug therapy of geriatric acute-care inpatients will be greater among intervention teams than control teams.

Qualitative data are not collected in confirmation or denial of a hypothesis, but in explanation of the findings of the intervention trial. These data will be used to understand staff perceptions of the effects of the intervention and the barriers and catalysts for its implementation.

### Outcomes: measurement

#### Readmission data

The Discharge Abstract Database (DAD) is managed by the Canadian Institute for Health Information. The DAD contains demographic, diagnostic, procedural and hospitalization data on hospital discharges (i.e., inpatient, acute, chronic, rehabilitation) and day surgeries in Canada. Data in the DAD can be used to identify dates of index hospital admissions and, subsequently, readmissions. A subset of the DAD relating to Ontario hospital admissions is held at the Institute for Clinical Evaluative Sciences (ICES) in Toronto, Ontario. ICES is an independent, non-profit organization with a core mission to conduct research that contributes to the effectiveness, quality, equity and efficiency of health care and health services in Ontario.

To develop a data file of hospital readmissions among patients discharged from CTTs participating in the SCRIPT Programme, we will use information in the DAD to establish a link between patients and the team that treated them. The key to using this method successfully lies in our identification of a patient's most-responsible physician. In Ontario, all practicing physicians are required to register with the province's regulatory body, the College of Physicians and Surgeons of Ontario (the CPSO). The CPSO assigns every registered physician a unique registration number. This number is a data field in the DAD; it appears on a discharged patient's record line. It is also publicly available with the physician's name on the Internet site of the CPSO.

Leaders of GIM divisions will provide us with the names and team assignments of physicians who were on rotation in GIM CTTs during SCRIPT's intervention periods. When we know a physician's name from GIM records, we can search for his or her profile in the CPSO Internet site. Physicians' CPSO registration numbers will serve as the link between their positions in GIM CTTs and discharge information in the DAD. We will compile a list of registration numbers of physicians who served in GIM during the study period, submit the list to database managers at ICES, and obtain a data set containing de-identified records of patients treated by a physician who served in GIM during the SCRIPT intervention period. In this way we will be able to determine the CTT where a patient received treatment.

The readmission dataset will be constructed with criteria similar to van Walraven et al. [20]. Patients will be identified as eligible for the readmission pool if they had a nonelective admission to one of the participating sites' GIM divisions through its medical or surgical department, were discharged alive from the participating site's GIM division in the same index admission, and had a nonelective medical or surgical readmission to any Ontario hospital within either 7 days or 30 days of discharge of the index admission. Patients will be excluded if they were admitted from or discharged to another hospital in the index admission; if they had no patient identifier or an invalid identifier; if they lacked key information; or if they exceeded the 99^th ^percentile of distributions of the following variables for all Ontario hospitals: hospital length of stay, number of previous hospitalizations, number of follow-up visits, or case resource consumption.

#### Length of stay data

Development of data for analysis of patients' length of stay is identical to the process for readmission data. Length of stay will be calculated as the number of days between the dates of the start and end of each admission. If both dates are the same then the length of stay will be 0 days.

#### Collaboration survey data

The purpose of the survey is to measure staff members' perceptions of interprofessional collaboration. It is an adaptation of the Collaboration Among Medical Staff Subscale (CMSS) from the Nurses' Opinion Questionnaire [21] The CMSS measures professional working relationships among nurses, physicians, and other health care professionals. It was developed on a belief that the organizational structure, role behavior, and communication patterns and methods between caregivers affect patient outcomes and staff satisfaction. Validity and reliability of data from published versions of the CMSS have been acceptable [22,23].

We will administer an adaptation of the CMSS containing 28 question items, fourteen unique items presented twice on one page, with vocabulary changed slightly to reflect the two major groups of GIM staff with whom a respondent works. Physicians will respond to survey items that target nurses, allied health staff, and unregulated clerical and clinical support workers; nurses respond to survey items targeting physicians, allied health staff, and unregulated clerical and clinical support workers; and allied health staff respond to survey items targeting physicians, nurses, and unregulated clerical and clinical support workers. The survey will be coded so that higher scores will represent higher perceived importance and existence of interprofessional collaboration within the team.

We plan three administrations of the survey instrument. The first will occur as close as possible to the first day of intervention implementation in the GIM teams. The second administration will occur after full implementation of the study's interventions, about eight weeks later. The third will occur eight weeks after the second.

In randomized trials, data from participants who are unaware of the past group condition, treatment or control, can be valuable [24,25]. Accordingly, the third survey administration will have particularly strong potential to survey staff who joined their GIM division and CTT after the conclusion of the intervention, eight weeks post-intervention. We expect that these staff members will be naïve to the past group condition of the clinical teams with which they are presently working.

In some of the hospital sites nursing and other health professionals' responsibilities require them to work on both intervention and control group teams; in these sites the survey instrument will direct survey respondents of these groups to self-select the team they work with most often from a complete list of the site's four GIM teams participating in the trial.

#### Patient satisfaction data

Patient satisfaction survey data is collected from post-discharge surveys of consenting Ontario hospital patients. Surveying is performed by the NRC+Picker company on behalf of a partnership initiative between the Ontario Hospital Association (OHA) and the Ontario Ministry of Health and Long-Term Care. Most of these data are analyzed and consolidated for reports published on an annual basis by Ontario's Hospital Reports Research Collaborative. The initiative includes a major program to measure acute care inpatients' satisfaction with their hospital stays. These data are typically held in our hospital sites' offices of quality improvement or risk management.

We will identify individual question items and dimensions in the OHA/NRC+Picker reports with potential to be impacted by the effectiveness of interprofessional collaboration among GIM division staff. We will attempt to obtain frequency distributions of patients' individual-level responses to these questions from hospital databases. Some data may be aggregated; individual responses may not be available. In these instances we will attempt to obtain percentages of respondents reporting positive scores on the question items and dimensions. Patient satisfaction data can be sorted and filtered into team-level groupings and can be accessed over the Internet at some of the participating hospitals sites. At other sites we will require assistance from hospital administrative functions to achieve linkages between responses and the teams where patients were treated.

#### Paging activity

For investigations of paging activity, potential subjects are GIM staff members who carry and use a hospital paging device. Development of a paging activity dataset will require assistance from two key resources: (1) GIM administrative functions that can identify GIM team members who carry pagers, and (2) reporting functions in a hospital site's information and communications technology division that can generate analytic reports of pages placed to GIM team members.

Administrative assistants in the hospital sites' Departments of Medicine will provide on-call schedules for medical staff members. On-call medical staff members carry pagers to provide coverage in various positions in the Department of Medicine, including GIM. Patient care managers in GIM teams will identify for us members of their team's nursing and allied health staff who carry paging devices.

After we have obtained the required information to identify individuals and positions in GIM that carry pagers, we will approach information technology reporting functions with the information. When we supply titles of GIM positions that carry pagers and paging identification numbers assigned to them, information technology staff is able to generate reports that can be used to determine the number of pages received by a specific paging identification number, and some other important characteristics of pager calls, chiefly, their designation as urgent or normal.

Recently we undertook exploratory conversations with members of two hospital sites' information technology divisions. Our goal was to probe the general character of paging records (i.e., data fields, content, frequency) that might be available for research use. These conversations suggested two key factors for consideration. First, while the nature of the request and the use of the data or reports in which the data routinely appear would be unusual, it is possible to generate time-delimited reports of activity on a selectively targeted group of paging devices. The second factor we learned is that there is a possibility that calls to paging devices can contain patient health information or other identifying information, and this information could also appear in administrative records of paging activity.

We appreciate the need to grant supreme confidentiality to both patient information and non-patient-related thoughts and expressions that staff members might convey to one another in their paging activity. For these reasons we will limit our requests to include only the minimum essential amount and character of information required to test our hypotheses. In particular, we will request that staff of the sites' information technology departments *remove/exclude *all personal health information and patient identifying information from any records and reports that are provided to us. This would include text that could appear in records relating to paging devices with alphanumeric capability.

We will request that information technology staff members *include *in records or reports: the number of the device receiving the page, the role/function to which it is assigned (e.g., Patient Care Manager, Occupational Therapist, Attending Staff Physician), the date and time the page was received by the device, the urgent/non-urgent status associated with the page and, if possible, the number and role/function which placed the call to the paging device.

#### Prescription drug therapy

We will examine an outcome measure originating in the evidence-based medicine paradigm. Clinical evidence-based guidelines exist [26] to support appropriate uses of drugs for treatment of diseases commonly seen in GIM patients: diabetes, community acquired pneumonia, congestive heart failure, hypertension, asthma, chronic obstructive pulmonary disorder, stroke, and acute myocardial infarction. A database exists that can be used for investigation of adherence to evidence-based prescribing practices. This database is used in the Ontario Drug Benefit (ODB) program.

For defined groups of individuals, the Ontario Ministry of Health and Long-Term Care covers most of the cost of prescription drug products listed in the ODB formulary. Importantly for our purposes, one of these groups is Ontario residents 65 years of age and older. For drug products prescribed by an authorized Ontario prescriber, the ODB program covers approximately 3,000 prescription drug products.

The ODB program pays for listed drug products for individuals eligible for ODB coverage if the drugs are purchased in an Ontario pharmacy that is on-line with the ministry's Health Network or from an Ontario doctor licensed to sell prescription drug products. In 2004–2005, 70% of ODB beneficiaries were age 65 or older. It is likely that a majority of patients admitted into and discharged from GIM wards in the participating hospital sites are in this age group. We believe that many are eligible beneficiaries under the ODB program and that a large share of their post-65 prescription drug use history will exist in databases of the ODB program.

For analyses of prescription drug therapy, subjects are patients who are admitted to GIM divisions of participating hospital sites on or after the start date of the intervention at the site, and who are discharged to home from the GIM division on or before the last day of the next full month that follows the conclusion date of the intervention period. We will construct an ODB record for these patients that extends for 60 days after their date of discharge.

### Sample size

Sample size and power were estimated with PASS software [27]. The trial's primary outcome is the proportion of unplanned hospital readmissions among patients discharged from a GIM team. We assume an intracluster correlation coefficient of 0.01. In this trial we expect there will be in excess of 200 patients admitted into each clinical teaching team in the 2-month intervention period. In total there will about 4000 patients admitted into and discharged from twenty clusters (teams) over five hospital sites. We assume that a typical rate of readmission among GIM patients within 30 days after discharge is ten percent. When significance is set at p < 0.05, the trial would have 93% power to detect a 5.0 percentage-point lower rate of unplanned hospital readmission among patients discharged from intervention CTTs than control CTTs (i.e., 10% versus 5%). A 5.0 percentage-point difference would represent a fifty-percent difference in readmission rates between intervention and control team patients.

If the rate of readmission within 30 days of discharge from GIM treatment is five percent, and if the intracluster correlation coefficient is 0.01, then the trial has 67% power to detect a 2.5 percentage-point lower rate of readmission among patients discharged from intervention teams. Again, this is a fifty-percent difference (i.e., 5% versus 2.5%).

### Randomization

#### Sequence generation

The trial is a cluster randomized trial. Stratification is by hospital site. Each stratum has exactly four GIM clinical teaching teams. In five hospital sites, each of four GIM clinical teaching teams will be randomized to intervention or control group status using numbers generated at random by the web site [28], a website service for generating random numbers. Two clinical teaching teams will be intervention teams; two others will be control teams. Assignment of teams is not performed in 'batch mode.' In other words, each unique hospital site's four teams will be assigned a group status with just a few months' lead time, usually after the conclusion of the intervention period at a previous site.

#### Allocation concealment

The unit of allocation will be the cluster, the GIM clinical teaching team. Concealed allocation of teams to treatment statuses will not be possible. SCRIPT Programme research staff will be required to know which teams are assigned to intervention status because researchers will need to work closely with intervention team leaders to develop intervention training regimes and fieldwork observation schedules.

#### Implementation

Names of GIM teams will be collected from hospital sites. These are names like Team A, Team B, Team C, and Team D. To assign the teams to intervention and control group statuses we will use the method described at the Internet site called Random.org [28].

A project research assistant will list the names of a site's four GIM teams in rows of a spreadsheet, or simply on a piece of paper. At the web site , we specify boundaries of a number sequence containing four numbers. The smallest number is 1; the largest is 4. Two numbers will be designated to correspond to intervention teams, e.g., numbers 1 and 2. Numbers 1 through 4 are returned by the site's random sequence generator, arrayed in one column of four rows, one number per row. This columnar sequence is copied into a spreadsheet column to the immediate right of the column holding the names of a hospital site's four GIM teams. After copying this column, the names of teams that appear to the left of, e.g., numbers 1 and 2, will be assigned to implement the intervention.

#### Blinding

Intervention administrators (i.e., core project staff) will not be blinded to team intervention assignments. Group assignments associated with specific outcomes data can, perhaps, be masked, and will be masked to the greatest extent possible. For example we believe that intervention assignments associated with data for readmission rates, lengths of stay, and use of optimal drug therapy can be concealed from the project's consulting statistician.

For other outcomes data, blinding will be difficult to achieve because these data will be managed by SCRIPT Programme staff who are closely associated with the intervention implementation. These are data relating to staff perceptions of interprofessional collaboration, patient satisfaction, and paging activity.

### Statistical methods

Team and patient outcomes will be compared on an intent-to-treat basis.

#### Readmission

Two patient follow-up periods will be investigated for unplanned hospital readmission: the primary outcome, 30 days post-discharge, and a secondary outcome period of seven days post-discharge. Discharge records will be examined for patients discharged to home between one week after intervention period commencement and one month after intervention conclusion. Readmission proportions will be calculated for patients discharged from intervention and control group teams. The significance of the difference in proportions will be tested with chi-square and association statistics adjusted for the cluster randomized design [29-32]. The primary aim of the trial is to estimate the population-level intervention effect on binary-coded readmission outcomes; consequently, generalized estimating equations will be employed to control for clustering.

Some patients could have multiple hospital admissions, possibly to different hospitals during the study period. Furthermore, some of the admissions may qualify as an "outcome" while others do not. This situation raises a possibility that patients could change clusters (teams) during the trial, by being readmitted to a different GIM team within the same hospital that discharged them earlier in the intervention period. Therefore, a patient will contribute an observation to the analysis only from their first admission during the intervention period. If the readmission is to a different hospital than the index admission, then the readmission is 'credited' to the hospital of the index admission.

#### Length of stay

Mean lengths of stay will be compared between patients discharged from intervention and control group CTTs. Intervention group effects will be estimated with linear mixed models.

#### Collaboration survey data

Confirmatory factor analytic methods will be used to investigate scale structure and reliability of data. Intervention group effects will be estimated with linear mixed models. Secondary analyses will investigate stability and change (growth) in survey scores over time.

#### Patient satisfaction

Survey data on patient satisfaction are available in a form that reports the percentage of patient respondents who provided a positive response to a survey question item. Further, responses are available for individual question items and for 'dimensions,' related question items aggregated into groups that probe a particular concept or construct. Rates of positive responses from intervention and control team patients will be tabulated; measures of association will be calculated; generalized estimating equations will adjust for cluster randomization.

#### Paging activity

Recent research has suggested that hospital paging systems can be problematic. Paging activity can be highly interruptive. Calls to staff are often clinically inappropriate or not urgent enough to justify a necessary quick callback [33]. Paging can be disruptive to patient care [34] and scheduled rounds [35,36]. Disruptive pages may cause medical errors [37].

If the intervention is implemented correctly, face-to-face communication could increase in frequency and effectiveness. By implication, we suggest that there is potential for reductions in paging activity within and between members of GIM professions.

Administrative records will permit us to examine the total number of pages placed to paging devices of staff members of intervention and control teams, and the number of pages marked as urgent. Numbers of pages to intervention or control teams will be standardized by the number of individuals who carry pagers in the groups. Intervention-control group differences will be examined with linear mixed models.

#### Prescription drug therapy

Our hypotheses about clinical indications and prescription drug use in GIM are motivated by beliefs that communicative and collaborative improvements in health care can be related to optimal drug therapy. There are four hypotheses to be tested. First, 'is continuity of patient drug therapy greater among patients of intervention teams than control teams?' This question can be investigated by determining whether a patient fills a prescription after discharge for a drug that was used before GIM admission.

A second hypothesis will examine whether admitted patients received evidence-indicated drugs for every diagnosis they were known to have while receiving care in GIM teams. The third and fourth hypotheses are subsets of the second. The third hypothesis focuses specifically on problems that were not responsible causes for admission to the GIM division. We suggest that drug therapies for these problems will not be physicians' foremost priorities in the care plan; hence, improvements in interprofessional care could be most likely to be detected in increased rates of drug therapies for these problems. The fourth hypothesis will investigate uses of drugs that are contraindicated among elderly patients [26]. Usually, GIM patients are elderly.

Response variables related to the hypotheses will binary coded. Effects of intervention and control team statuses will be compared with generalized estimating equations.

#### Covariates

Model-based estimates of intervention effects on the outcomes of readmission, length of stay, use of optimal drug therapy, patient satisfaction, and use of paging devices will employ patient-level and team-level covariates. Patient-related factors to be controlled will be patients' age and gender.

Other covariates will be measures of hospital health care processes hypothesized to be related either to outcome measures or to intervention uptake. The intervention's focal concern is the improvement of interprofessional performance of GIM teaching teams. Therefore, we propose to use the following constructs as structural indicators of functional team processes of care:

• *Ward-based assignment of medical, nursing, and allied health professional staff*. 'Ward basing' refers to hospital practices specifying the deployment of healthcare staff members to a single physical ward location. This arrangement is in contrast to assignments that have staff members working among different medical teams or nursing units as they treat patients. For example, if nurses and nursing units are fixed to physical ward locations, then medical team and professional health staff members could be assigned to patients in such a way that they are required to work with members of several different nursing units. If so, they are said to 'cross teams or units.' On the other hand, if medical teams are fixed to physical ward locations, then it is possible that nurses and professional health staff could be required to work among multiple different medical teams. It is also possible that both medical teams and nursing units are given fixed assignments to single ward locations and professional health staff are assigned to work across multiple combinations of medical teams and nursing units. All of these configurations create variation in the degree to which GIM staff are consolidated to work in one physical location.

Work assignments that see GIM staff consistently – and as a matter of hospital policy – working in multiple physical locations, where work colleagues are more likely than not to be unfamiliar, might have negative impacts on achievement of effective interprofessional collaboration and patient outcomes. An indicator variable will be developed to code whether clinical teaching teams are organized to consolidate interprofessional teams and team members to a single physical ward location.

• *Team-dedicated discharge planner/coordinator*. GIM divisions in some TAHSN hospital sites utilize identified individuals in discharge planning roles as Discharge Planners or Discharge Coordinators. In other sites the discharge coordination function is a responsibility of licensed social workers. We suggest both that patient outcomes with respect to readmission and length of stay, and increased team collaboration and coordination, are influenced positively when the role of discharge planning is performed by an individual with dedicated, team-focused discharge responsibilities (e.g., a registered nurse) instead of an individual with discipline-specific responsibilities (e.g., a social worker). Measured at the level of the clinical teaching team, this construct will be a binary coding of whether the team employs a full-time discharge planner (typically a registered nurse), a social worker with additional responsibilities of coordinating discharge planning, or another individual.

• *Length of regularly scheduled meeting time for interdisciplinary rounds, per team*. We define an 'interdisciplinary round' as a patient-focused meeting that occurs at least one day per week (Monday to Friday), every week, where regular attendance is expected by a team resident physician and leaders of two other clinical professions, nursing and allied health professional staff. The variable will be measured at the level of the clinical teaching team, operationalized as average (mean) meeting hours per week during the time the intervention is implemented at the hospital site.

• *Nursing staff characteristics*. Measures will be developed to adjust for team-level variation in nurse staffing. It is expected that hospital administrative data will be available to enable development of measures of nursing staff characteristics such as: (1) the mean of the daily proportion of registered nurses in a team's staffing mix while the intervention was in progress, or (2) the mean of the daily proportion of nurses with baccalaureate degrees who worked on a team while the intervention was in progress, and (3) the proportion of a team's nursing hours performed by registered nurses per unit of time, or (4) the proportion of a team's nursing hours performed by baccalaureate-prepared nurses per unit of time.

• *Nursing workload measures*. Data are expected to be available to measure team-level variation in nursing workload. Measures will be continuous variables and could include: (1) the patient-to-nurse ratio per unit of time during the intervention period, per team; (2) the mean number of hours of nursing care worked on a team, per patient-day during the intervention period; or (3) nurses' acuity-adjusted patient load per unit of time during the intervention, per team.

#### Qualitative data

Observational notes and interviews will be analyzed with a thematic inductive approach, whereby meanings emerge from the data and are grouped into key themes and sub-themes [38]. The research team will discuss and negotiate the emerging analyses to help improve conceptual understanding, and reach agreement on interpretation. The data will be analyzed from the perspectives of intervention impact and staff behavioural and perceptual changes over time. Interaction frequencies will be analyzed to examine intervention uptake.

#### Potential benefits

The SCRIPT intervention was developed with a goal of increasing interprofessional collaborative communication. It is expected that strong collaborative communication is a factor in achieving (i) better patient health outcomes in shorter times, (ii) higher reported patient satisfaction with the experience in the GIM department, and (iii) improvements to the hospital work environment gained through perceptions of increased commitment to collaboration and fewer interruptive communications.

If analyses find that use of evidence-based prescription drug therapy is more likely or more abundant among intervention teams than control teams, then a range of benefits are possible. For example, satisfactory ability to manage a patient's disease could be increased, complications and length of stay could be reduced, and discharge could be made more rapidly. Moreover, if there is a general causal relationship between sub-optimal prescription drug therapy and subsequent unplanned readmission to hospital, investigations into prescription drug use could inform remedial goals of reducing drug-related unplanned readmissions.

#### Potential harms

Potential for harms is minimal. The trial poses low risk because it requires no contact between patients and the research team, and because the intervention does not impose major changes on health care processes. There is no expectation that participants from whom data are collected for any of the quantitative outcome measures will face any harm.

A privacy-related concern surrounding use of data relating to prescription drug therapy involves our pursuit of a dataset that could be used to identify GIM teams where patients' prescription drug therapy was shown to be sub-optimal or not regularly in accord with evidence-based best practices. We recognize that it is necessary to protect confidentiality of GIM members whose roles give them responsibility for influencing segments of GIM patients' prescription drug therapy. Data and analyses used in these investigations will be stored and disseminated with great sensitivity.

At the same time, given the evidence base that now exists to help guide appropriate therapeutic use of prescription drugs, an investigation of prescription drug therapy as an outcome measure in an intervention study is a highly important and legitimate research endeavour.

Confidentiality of individual, patient-level records relating to hospital readmissions, lengths of stay, and prescription drug therapy is governed by policies and procedures of the Institute for Clinical Evaluative Sciences. ICES policies are both strict and effective. These data records can not be removed on any storage or transport medium from the physical ICES premises. ICES data facilities employ stand-alone data servers that are not connected to the Internet.

## Discussion

Despite continuing international interest in improving interprofessional collaboration and service delivery in health care [2,3], the evidence base for interventions is inconclusive [6,8]. This cluster randomized controlled trial is the first study that will explicitly design and rigorously evaluate a front-line intervention on interprofessional collaborative communication to support patient-centred care.

Previous research has indicated that well-coordinated interprofessional collaboration is difficult to achieve in fast-paced acute care hospital environments [39,40]. Given this situation, the design of an effective intervention requires care and attention to important contextual factors. To ensure the intervention will be both sufficiently sensitive and robust to operate within such environments, as outlined above, we undertook a series of preliminary qualitative observations within the study sites.

An important finding from our qualitative data is that opportunities for interprofessional collaborative communication occur during informal, unplanned interactions, outside of formal structured meetings. These opportunistic interactions can be highly consequential: they are initiated by professionals many times during a working day, usually to obtain information from another professional who is sharing responsibility for the same patient, and/or to impart information to him or her about the patient. Analysis has shown that three core elements of communication are typically absent from such encounters: named self-introduction and description of role with respect to the patient under discussion; sharing of planned activities for the patient; and elicitation of the counterpart's point of view. We drew upon these findings to design an intervention for creating a culture of interprofessional communication where the fundamentals of collaboration are a routine and normalized part of opportunistic, informal encounters.

Our outcome measures are well-suited to measuring the intervention's impact, because they are measures with large, motivated, and interested constituencies: front line hospital staff, hospital managers, and patients. Two outcome measures in particular – paging frequency and continuity of pre- and post-hospital drug therapy – can help investigate novel hypotheses bearing on the complexities and consequences of interprofessional collaborative communication. They have excellent potential to redress existing clinical and community problems in interprofessional team communication.

## Abbreviations

CMSS – Collaboration among Medical Staff Subscale

CPSO – College of Physicians and Surgeons of Ontario

CTT – Clinical teaching team

DAD – Discharge Abstract Database

GIM – General internal medicine

ICES – Institute for Clinical Evaluative Sciences

IECPCP – Interprofessional education for collaborative patient-centred practice

LOS – Length of stay

ODB – Ontario Drug Benefit

OHA – Ontario Hospital Association

PASS – Power Analysis and Sample Size

PGY*n *– Post-graduate year *n*

RCT – Randomized controlled trial

SCRIPT – Structuring Communication Relationships for Interprofessional Teamwork

TAHSN – Toronto Academic Health Sciences Network

UK – United Kingdom

## Competing interests

The author(s) declare that they have no competing interests.

## Authors' contributions

*MZ *participated in writing the protocol, applying financial support, approval of ethical committees, intervention design and is the principal investigator for this study.

*SR *participated in writing the protocol, intervention design and is a co-investigator with expertise in qualitative methods.

*AR *participated in writing the protocol, approval of ethical committees, qualitative data collection and analysis, intervention design and is the co-ordinator for the trial.

*CK *participated in writing the protocol, approval of ethical committees, intervention design and is co-ordinating the quantitative data collection/analysis for the trial.

*LGC *participated in writing the protocol, approval of ethical committees, intervention design, and qualitative data collection and analysis.

*KLM *participated in writing the protocol, and is involved in collection and analysis of qualitative data for the trial.

*LL *participated in applying financial support and is a co-investigator with expertise in qualitative methods.

*KT *participated in writing the protocol and is leading the quantitative data collection/analysis for the trial.

All authors read and approved the final manuscript.
